# 3D path planning for robot-assisted vertebroplasty from arbitrary Bi-plane X-ray via differentiable rendering

**DOI:** 10.3389/frobt.2026.1759366

**Published:** 2026-02-26

**Authors:** Blanca Inigo, Benjamin D. Killeen, Rebecca Choi, Michelle Song, Ali Uneri, Majid Khan, Christopher Bailey, Axel Krieger, Mathias Unberath

**Affiliations:** 1 Johns Hopkins Whiting School of Engineering, Baltimore, MD, United States; 2 Johns Hopkins School of Medicine, Baltimore, MD, United States

**Keywords:** artificial intelligence, computer-assisted interventions, deep learning, fluoroscopy, intraoperative planning, spine surgery

## Abstract

Robotic systems are transforming image-guided interventions by enhancing accuracy and minimizing radiation exposure. A significant challenge in robotic assistance lies in surgical path planning, which often relies on the registration of intraoperative 2D images with preoperative 3D CT scans. This requirement can be burdensome and costly, particularly in procedures like vertebroplasty, where preoperative CT scans are not routinely performed. To address this issue, we introduce a differentiable rendering-based framework for 3D transpedicular path planning utilizing bi-planar 2D X-rays. Our method integrates differentiable rendering with a vertebral atlas generated through a Statistical Shape Model (SSM) and employs a learned similarity loss to refine the SSM shape and pose dynamically, independent of fixed imaging geometries. We evaluated our framework in two stages: first, through vertebral reconstruction from orthogonal X-rays for benchmarking, and second, via clinician-in-the-loop path planning using arbitrary-view X-rays. Our results indicate that our method outperformed a normalized cross-correlation baseline in reconstruction metrics (DICE: 0.75 vs. 0.65) and achieved comparable performance to the state-of-the-art model ReVerteR (DICE: 0.77), while maintaining generalization to arbitrary views. Success rates for bipedicular planning reached 82% with synthetic data and 75% with cadaver data, exceeding the 66% and 31% rates of a 2D-to-3D baseline, respectively. In conclusion, our framework demonstrates the feasibility of versatile, CT-free 3D path planning for robot-assisted vertebroplasty, accommodating diverse intraoperative imaging conditions without requiring preoperative CT scans.

## 1 Introduction

Robotic systems are transforming the Operating Room (OR) by improving surgical outcomes, increasing safety, and reducing radiation exposure through remote actuation and fewer fluoroscopy acquisitions. A key component of robotic automation is accurate surgical path planning, which typically requires detailed 3D anatomical information. For complex spinal procedures, this information is obtained through preoperative CT scans that can be registered with intraoperative imaging to guide the robot. However, for less severe conditions such as vertebral compression fractures, pre-operative CTs are not available as they are not needed to diagnose the conditions. For these procedures, acquiring a preoperative CT scan is impractical due to increased time, cost, and radiation exposure. As a result, high-volume yet relatively straightforward procedures, like vertebroplasty, have seen limited benefit from robotic assistance. Developing methods that enable accurate surgical planning using just a few 2D X-ray images could make robotic assistance viable for these cases.

Over the past decade, deep learning models have shown potential for reconstructing 3D anatomy from 2D images, potentially eliminating the need for CT scans. However, these models often operate under strict assumptions, such as the availability of standardized imaging angles or minimal relative tilt between views (Kyung et al., 2023; Chen et al., 2023; Saravi et al., 2023; Chen et al., 2024). These constraints limit their applicability in real-world clinical settings, where imaging conditions vary widely and the most informative views are often patient-specific (Killeen et al., 2023a). Additionally, many of these models act as “black boxes,” lacking interpretability and explicit anatomical constraints that are particularly concerning in high-stakes applications such as spine surgery.

Vertebroplasty—a minimally invasive procedure where a needle is carefully inserted through the pedicle into the vertebral body to inject bone cement—could benefit from robotic assistance to improve safety and efficacy. However, existing systems are often bulky, costly, and disruptive to clinical workflows, leading to limited adoption, particularly in low-volume settings (D’Souza et al., 2019).

To overcome these limitations, we present *Spine-DART* (Differentiable Atlas-based Reconstruction and Trajectory planning for the Spine), a needle trajectory planning strategy that uses intraoperative 2D X-ray images to generate accurate 3D guidance without requiring a preoperative CT scan. Our optimization-based framework performs end-to-end 3D transpedicular path planning from two arbitrary X-rays. By combining differentiable rendering with a vertebral Statistical Shape Model (SSM) and a learned similarity loss, the method jointly optimizes the SSM’s pose and shape without assuming fixed imaging geometry. This allows for anatomically plausible shape constraints and consistent point correspondences, enabling effective alignment of 3D anatomy and planned paths with the input X-rays.

Unlike existing deep learning methods that rely on fixed imaging conditions, Spine-DART adapts to the variability of real-world intraoperative fluoroscopy, enabling generalizable 3D planning from arbitrary views. While atlas-based pipelines offer valuable anatomical priors, they often depend on hand-crafted similarity metrics and non-differentiable optimization procedures that are sensitive to initialization and prone to failure without manual intervention. These limitations hinder their robustness, scalability, and integration into automated systems.

Spine-DART overcomes these challenges by integrating statistical shape models with deep learning. By combining anatomical priors with a learned similarity loss and differentiable rendering, our framework supports gradient-based optimization of shape and pose, yielding anatomically consistent reconstructions without requiring preoperative CT or constrained X-ray geometries. This hybrid approach balances interpretability with flexibility, enabling accurate and reliable CT-free 3D path planning in unconstrained intraoperative settings.

By eliminating the need for CT and integrating seamlessly into current clinical workflows, Spine-DART enhances surgical planning precision and supports the broader adoption of compact, cost-effective robotic platforms for procedures like vertebroplasty (Opfermann et al., 2021; Song et al., 2025).

## 2 Related work

3D surgical path planning, particularly for transpedicular access, has traditionally relied on preoperative CT-based methods, which provide detailed anatomical models for direct 3D planning and integration with navigation systems (Otomo et al., 2022; Qi et al., 2022; Singh et al., 2020; Wang et al., 2024). In intraoperative scenarios where CT is unavailable or impractical, alternative strategies have emerged. These can be broadly categorized as: (1) direct 2D path planning followed by triangulation or interpolation to recover a 3D trajectory (Killeen et al., 2023b; Naik et al., 2022), and (2) 3D anatomical reconstruction from intraoperative 2D images followed by path planning (Demin et al., 2024; Shiode et al., 2021; Uneri et al., 2014). While both approaches eliminate the need for preoperative imaging, they present trade-offs in terms of accuracy, sensitivity to imaging geometry, workflow integration, and interpretability. Our method extends the second category by enabling 3D vertebral reconstruction and automatic path planning from any two fluoroscopic viewpoints without requiring known geometries or standardized angles.

The first category, 2D path planning with 3D interpolation, involves annotating surgical paths directly on fluoroscopic images and then reconstructing the 3D trajectory via triangulation. Killeen et al. (2023a) demonstrated this technique using fluoroscopy images for pelvic screw fixation. While this method is straightforward and computationally efficient, the 2D predictions are made independently for each view, without considering their correlation through the underlying 3D path. This can result in misaligned annotations that reduce the accuracy of the final surgical plan.

The second category reconstructs 3D anatomy from 2D images, allowing path planning directly in 3D space. Deep learning (DL) methods have shown promise in this area for their reconstruction accuracy and inference speed (Chen et al., 2024). Approaches by Kyung et al. (2023), Chen et al. (2023), and Ying et al. (2019) require paired orthogonal X-rays (e.g., AP and lateral) to constrain the reconstruction, limiting their flexibility in unconstrained intraoperative settings. Furthermore, most methods are trained and tested on Digitally Reconstructed Radiographs (DRRs), which can lead to generalization challenges when applied to real X-ray images.

More flexible multi-view formulations have been proposed to relax strict view constraints. Jecklin et al. (2022) introduced a method for intraoperative vertebral shape reconstruction from multiple sparse X-ray views, allowing greater freedom in view selection compared to paired orthogonal approaches. However, this method was validated using a relatively large number of input images (four or eight) acquired under partially constrained view geometries rather than fully arbitrary intraoperative poses. To address the domain gap between synthetic DRRs and real fluoroscopic images, Jecklin et al. (2024) further proposed domain adaptation strategies based on style transfer to improve robustness on real X-ray data. Finally, Gopalakrishnan et al. (2024) presented a differentiable X-ray rendering framework for 2D–3D image registration, closely related in spirit to reconstruction-based planning approaches; however, it assumes access to a patient-specific 3D anatomical model, which is not available in the intraoperative workflow considered in this work.

Atlas-based methods offer an alternative by deforming a Statistical Shape Model to match input images. These ensure anatomical plausibility and enable automatic propagation of annotations (Vijayan R. C. et al., 2019; Han et al., 2019; Lamecker et al., 2006; Ehlke, 2020). However, traditional atlas-based registration pipelines typically rely on conventional optimization techniques, which are sensitive to initialization and often exhibit a limited capture range. As a result, they generally require semi-manual interaction to ensure convergence to anatomically valid solutions, limiting their scalability in time-sensitive surgical settings (Vijayan R. et al., 2019, Vijayan R. C. et al., 2019; Yousefi et al., 2011; Duay et al., 2008). Moreover, their non-differentiable components pose challenges for integration into modern learning-based frameworks.

Spine-DART addresses these challenges by combining atlas priors with deep learning, preserving the anatomical plausibility and interpretability of SSMs while overcoming the limitations of conventional optimization. By incorporating differentiable rendering and a learned similarity loss, Spine-DART enables joint optimization of shape and pose through gradient-based methods. This framework supports robust 3D path planning from arbitrary X-ray viewpoints without relying on patient CT or constrained imaging geometry, while also maintaining interpretability through intermediate reconstruction and planning steps.

## 3 Methods

**FIGURE 1:**
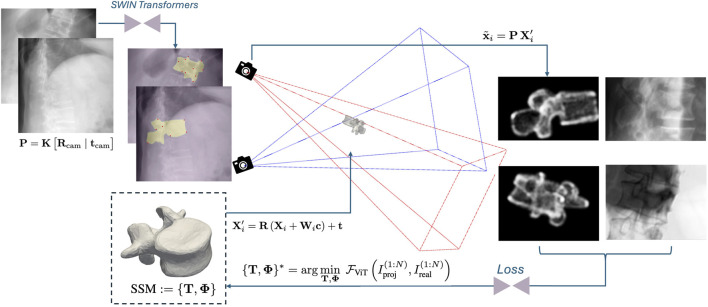
Proposed pipeline overview: Two SWIN transformers analyze X-ray images to detect and segment vertebrae, identifying landmarks to initialize the SSM’s pose. Gaussian splatting is used to render SSM projections in optimization steps. A multi-view Vision Transformer (ViT) calculates a similarity loss from these projections and original X-rays, refining the SSM’s pose and shape for accurate 3D vertebral reconstruction with consistent point correspondences.

Spine-DART enables 3D vertebral reconstruction and automatic transpedicular path planning directly from biplanar 2D X-ray images (Figure 1). It consists of four main components:

1. **Vertebral SSMs** that act as shape priors, with their pose and shape parameters optimized during registration.
2. **DL networks** that extract semantic features from X-rays to initialize the SSM pose.
3. **A differentiable rendering pipeline** based on Gaussian splatting that generates vertebral projections while supporting gradient-based optimization.
4. **A multi-view learned similarity loss** between the rendered projections and the input X-rays.

Since the vertebral SSM is pre-annotated with the transpedicular trajectory, the final surgical path plan is inherently transferred to the registered SSM during optimization.

### 3.1 Vertebral statistical shape model

Statistical Shape Models (SSMs) of anatomical structures have long supported surgical planning and automated image annotation (Killeen et al., 2023b; Ambellan et al., 2019). In our approach, SSMs serve two key purposes: they reduce optimization complexity by constraining the solution space to anatomically plausible vertebral shapes, and they enable direct propagation of anatomical annotations—such as transpedicular trajectories—through consistent point correspondences. Given the substantial anatomical variability along the spine, we follow common practice and construct region-specific SSMs for different vertebral groups to improve both specificity and anatomical realism (Panjabi and White, 1980; Bredbenner et al., 2014; Wai et al., 2023).

Accordingly, we developed four distinct SSMs: (1) T1–T4, (2) T5–T8, (3) T9–T12, and (4) L1–L5, using vertebral segmentations from the CTSpine1K dataset (Deng et al., 2023) while excluding subjects from the VerSe dataset (Sekuboyina et al., 2021). SSM construction followed a two-stage registration and modeling pipeline. Rigid registration was performed using Generalized Procrustes Analysis (Gower, 1975) to remove global translation, rotation, and scale differences, followed by Iterative Closest Point (ICP) (Zhang, 2021) to establish initial point correspondences across meshes. Deformable registration was initialized using non-rigid Coherent Point Drift (CPD), implemented via the pycpd library, to align all meshes to a reference shape and generate an initial SSM. The model was then iteratively refined by alternating ICP-based correspondence updates with Principal Component Analysis (PCA), optimizing the shape coefficients to capture anatomically plausible inter-subject variability while suppressing implausible deformations.

Each resulting SSM is parameterized by 15 principal modes of variation, which account for approximately 90% of the population-level shape variability. These shape parameters are jointly optimized with 6 pose degrees of freedom (3 for rotation and 3 for translation) during the reconstruction process.

### 3.2 Automatic X-ray annotation for semantic contextualization

**FIGURE 2:**
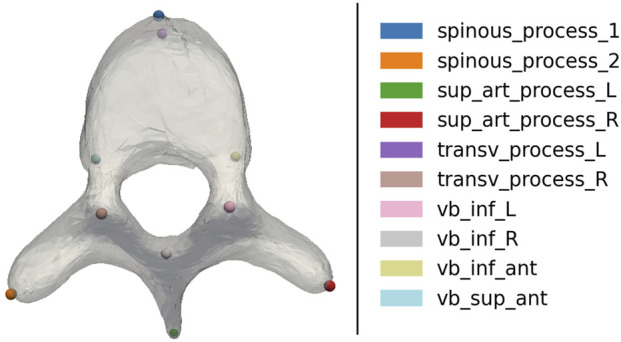
3D vertebral landmarks used for initialization of the SSM 3D pose.

Like most optimization-based methods, Spine-DART requires a reasonably accurate initialization of parameters. To achieve this, we trained two DL models with the same SWIN Transformer backbone (Liu et al., 2021) to perform vertebral semantic segmentation and landmark detection on X-ray images. Both models were trained using Digitally Reconstructed Radiographs (DRRs) generated under controlled conditions, with sufficient variability in imaging parameters and domain randomization to bridge the gap between synthetic and real X-rays (Gao et al., 2023). Additionally, we leveraged our SSMs to propagate 10 anatomical landmarks within the vertebral body and processes (Figure 2). To ensure high-quality supervision, we included only those vertebrae where the SSM achieved a registration DICE score above 85%, indicating a reliable fit.

Using DeepDRR (Unberath et al., 2018, Unberath et al., 2019; Gao et al., 2023), we generated a training dataset of over 150,000 DRRs, each paired with its corresponding 2D vertebral segmentations and keypoints.

### 3.3 Differentiable rendering for 3D vertebra reconstruction

Differentiable rendering simulates the generation of 2D images from 3D models while enabling gradient computation and backpropagation. This allows for the optimization of 3D parameters by comparing rendered projections to input X-rays. Our approach leverages this framework to iteratively optimize vertebral **shape** and **pose** parameters. Each component in the rendering pipeline is differentiable with respect to the 21 degrees of freedom (DoF), which are split into:

- **Rigid transformation** T={R,t} : 6 DoF, where R∈SO(3) is a rotation matrix and $t\in R^{3}$ is a translation vector.
- **Shape deformation** Φ : 15 DoF, parameterized by shape coefficients $c\in R^{15}$ associated with the principal modes of variation of the Statistical Shape Model (SSM).

We define the SSM parameterization as:

$$\text{SSM}:=\left\{T,\Phi \right\}$$

Each new shape is defined as a linear combination of the SSM shape basis followed by a rigid transformation. Let N denote the number of vertices in the canonical template mesh, which is shared across all shapes after registration and correspondence establishment. The SSM is constructed by stacking the registered training shapes into $R^{3}$ , computing the mean shape, and performing Principal Component Analysis (PCA) on the shape covariance. Retaining the top 15 principal components yields the shape basis matrix $W_{i}\in R^{3\times 15}$ , whose columns represent the dominant modes of anatomical variation.

Specifically, for each canonical vertex $X_{i}\in R^{3}$ , the transformed vertex is given by:

$$X_{i}^{\prime }=R\left(X_{i}+W_{i}c\right)+t$$

where $W_{i}\in R^{3\times 15}$ is the per-vertex block of the shape basis corresponding to vertex i, and $c\in R^{15}$ are the shape coefficients.

This formulation allows for continuous and differentiable shape deformation and pose transformation.

To render the transformed SSM in 2D, we use a CUDA-accelerated Gaussian Splatting renderer (Ye et al., 2024), which models each vertex as an anisotropic 3D Gaussian. Given a pinhole camera projection matrix:

$$P=K\left[\begin{array}{c} R_{\text{cam}}|t_{\text{cam}} \end{array}\right],$$

each transformed vertex $X_{i}^{\prime }\in R^{4}$ (in homogeneous coordinates) is projected into the image plane as:

$${\tilde{x}}_{i}=P\;X_{i}^{\prime },\;\text{and}\;x_{i}=\left(\frac{{\tilde{x}}_{i}}{{\tilde{z}}_{i}},\frac{{\tilde{y}}_{i}}{{\tilde{z}}_{i}}\right)$$

Gaussian Splatting renders the 2D projections $x_{i}$ as elliptical blobs, accounting for local surface orientation and perspective. This enables end-to-end differentiability and gradient flow through both shape and pose parameters.

To address discrepancies between real and generated projections due to differences in image formation, we introduce a learned similarity metric, $L_{\text{sim}}$ , based on a multi-view Vision Transformer (ViT) (Black and Souvenir, 2024), which is more robust than traditional metrics such as mutual information (MI) or normalized cross-correlation (NCC). The similarity loss is defined as:

$$L_{\text{sim}}=F_{\text{ViT}}\left(I_{\text{proj}}^{\left(1:N\right)},I_{\text{real}}^{\left(1:N\right)}\right)$$

where $I_{\text{proj}}^{\left(1:N\right)}$ and $I_{\text{real}}^{\left(1:N\right)}$ are the rendered and real X-ray images from N viewpoints (with N=2 in our setup), and $F_{\text{ViT}}$ encodes spatial and geometric consistency between views.

The goal is to find the optimal shape and pose parameters that minimize the similarity loss:

$${\left\{T,\Phi \right\}}^{*}=\arg\underset{T,\Phi }{\min}F_{\text{ViT}}\left(I_{\text{proj}}^{\left(1:N\right)},I_{\text{real}}^{\left(1:N\right)}\right)$$

This differentiable formulation enables end-to-end gradient-based optimization. The similarity loss $L_{\text{sim}}$ is backpropagated through the ViT, the Gaussian splatting renderer, and the SSM parameters, allowing iterative refinement of both vertebral shape Φ and pose T until the rendered projections align with the real X-rays.

### 3.4 Automatic transpedicular path planning

Transpedicular entry and exit points were manually annotated once on the canonical template mesh of each vertebral SSM by a student, following standard surgical guidelines for pedicle cannula placement. Because all shapes in the SSM share consistent vertex correspondence, these landmarks are defined in the SSM coordinate frame and are automatically transferred during reconstruction via the optimized shape and pose parameters. This results in a patient-specific transpedicular path that is anatomically consistent with the reconstructed vertebra, without requiring additional optimization or per-case manual annotation.

## 4 Experimental results

### 4.1 Dataset

The trainable components of Spine-DART—including the Swin Transformers for semantic segmentation and landmark detection, and the Vision Transformer (ViT) for the learned similarity metric—were trained on DRRs generated from two sources. First, we used 280^−ΔΔCT^ scans from the colon subset of the CTSpine1K dataset (Deng et al., 2024), selected for their relatively small slice thickness ( 0.88±0.16 mm) compared to the other CTSpine1K cohorts, which is important for generating high-fidelity DRRs that closely resemble real X-ray images. Second, we incorporated 97 high-resolution torso CT scans from the New Mexico Decedent Image Database (2025) to further enrich anatomical diversity and image resolution.

For evaluation, we used the VerSe-small dataset described in Chen et al. (2024) as our benchmark and compared our vertebra reconstruction results with the ReVerteR model. The final test set consisted of 140^−ΔΔCT^ scans comprising a total of 1,407 vertebrae.

We generated two DRR datasets (Unberath et al., 2018) from VerSe-small for evaluation:

1. *VerSe-small_ort*: A set containing only orthogonal views—Anteroposterior (AP) and Lateral (LAT)— to enable direct comparison with the reconstruction results reported by ReVerteR.
2. *VerSe-small_random*: A second set consisting of uniformly distributed DRRs, synthesized around the spinal axis of each scan to evaluate our method under more diverse view conditions.

In addition to synthetic evaluations, we tested our model on real X-ray data acquired from a cadaver study. For this experiment, we used a LoopX device to collect a CT scan and 35 fluoroscopic images from varying orientations. Ground-truth vertebra segmentations were obtained using TotalSegmentator (Wasserthal et al., 2023) with manual refinement to ensure accuracy. Table 1 shows an overview of the data used.

**TABLE 1:** Overview of datasets used for training and testing our DL models.

	Training		Testing	
	Colon	NMDID	VerSe-small	Specimen
CT	280	97	140	1
Vertebrae	4,036	1,530	1,407	16
DRR/X-ray	113,000	48,000	7,000	35

All our SSMs were created from the vertebra segmentations provided in the CTSpine1k dataset (Deng et al., 2024).

### 4.2 Automatic X-ray annotation models

**FIGURE 3:**
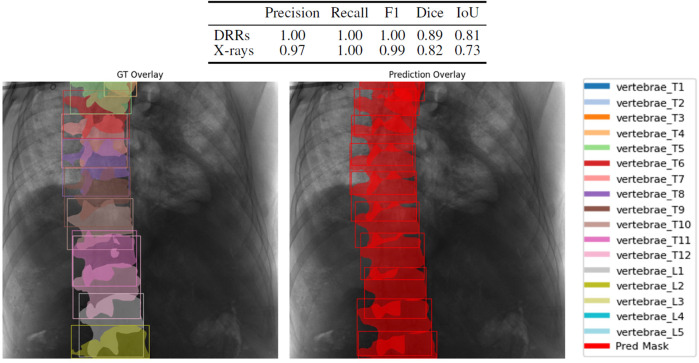
Top: segmentation performance metrics for synthetic DRRs and real X-rays. Right: qualitative vertebra segmentation result on a real X-ray.

**FIGURE 4:**
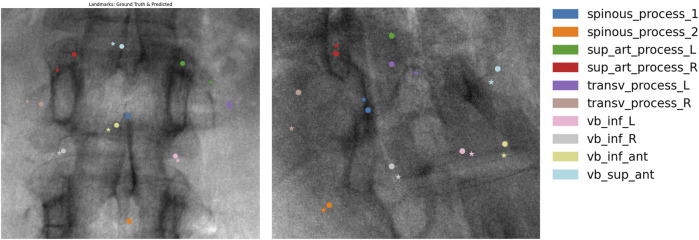
Qualitative results of our landmark detection model. Predictions (circles) and ground truths (stars) overlaid on real X-ray images.

**FIGURE 5:**
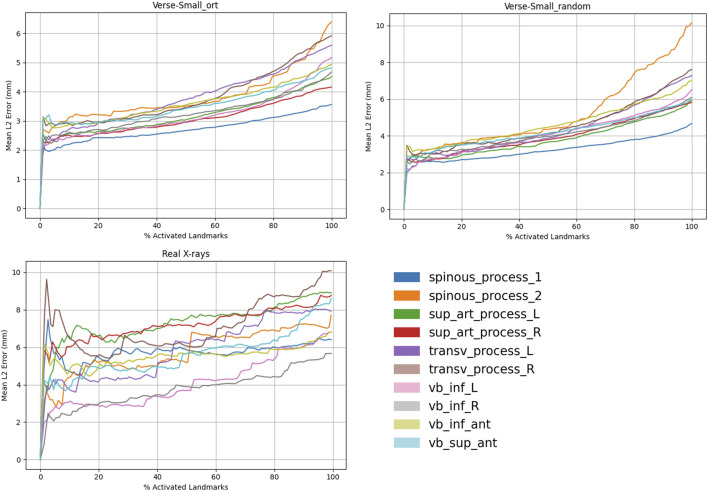
Landmark detection results across different domains. We report the mean Euclidean distance (mm) versus confidence percentile for orthogonal (AP/LAT) (left), random (middle), and real X-ray views (right).

We used a Mask R-CNN with a SWIN transformer backbone (Chen et al., 2019) to train a model on vertebra detection and semantic segmentation (Figure 3). The same backbone was retrained for landmark detection (Figure 4). Both models were evaluated on VerSe-small (orthogonal and random views) and on real X-rays. Figure 5 illustrates the 2D landmark localization error (in mm) as a function of the percentage of predicted activation peaks, determined by varying the detection threshold. As more landmarks are detected (i.e., higher percentages), the average localization error increases, reflecting a trade-off between detection sensitivity and precision.

### 4.3 3D vertebral reconstruction

We evaluated the accuracy of our 3D vertebra reconstruction pipeline on the VerSe-small dataset, which includes 140 spines and 1,407 vertebrae. Our approach was benchmarked against ReVerteR (Chen et al., 2024), a DL-based vertebra reconstruction model, using the same dataset and view protocol (AP/LAT) for fair comparison.

Each vertebra was reconstructed using two DRRs randomly selected from the synthetically generated AP and LAT views in the *VerSe-small_ort* set. Initialization was performed using vertebra detections and 2D landmarks predicted by our model, which defined the initial pose of the SSM. The reconstruction was then iteratively refined via gradient-based optimization, driven by the predicted Mean Average Surface Distance (MASD) between the rendered and real projections. This similarity loss, $L_{\text{sim}}$ , was computed using our multi-view ViT and backpropagated through the differentiable renderer to update shape and pose.

To accurately initialize the SSM pose, each pair of input views must contain at least three predicted landmarks in common to enable reliable 3D triangulation and subsequent alignment to the atlas. This condition is not always satisfied, which limits the initial reconstruction stage to 1,038 vertebrae. However, since the AP and LAT views follow fixed orientations, we can incorporate spatial priors to enable a fallback strategy. Specifically, we use a set of “backup landmarks” —including the vertebral centroid, superior, inferior, left, and right points—which allow us to initialize and reconstruct the remaining 369 vertebrae.

**FIGURE 6:**
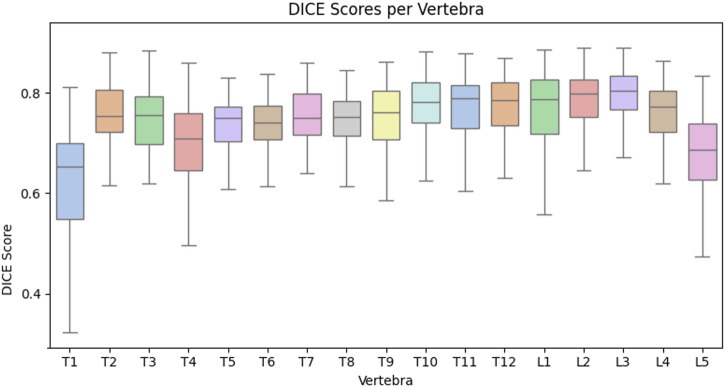
Per-vertebra reconstruction accuracy for Spine-DART on orthogonal-view synthetic data.

Figure 6 presents boxplots of the reconstruction Dice scores stratified by vertebral level, while Table 2 provides a quantitative comparison of Spine-DART against ReVerteR (Chen et al., 2024) and a baseline method based on normalized cross-correlation (NCC). While ReVerteR achieves the highest reconstruction accuracy, our optimization-based method remains competitive, outperforming the traditional NCC baseline by a large margin across all metrics. Notably, our framework offers several key advantages: it generalizes to arbitrary input views without retraining, supports integration with gradient-based priors and constraints, and maintains interpretability through its use of an SSM. This enables direct point correspondences with anatomical atlases and facilitates downstream tasks such as surgical planning or semantic labeling.

**TABLE 2:** Quantitative comparison of vertebra reconstruction accuracy across models given orthogonal-view synthetic inputs. Metrics reported are DICE, Normalized Surface Dice (NSD), and 95th percentile Hausdorff Distance (HD-95).

Model	DICE ↑	NSD ↑	HD-95 ↓
ReVerteR Chen et al. (2024)	0.7685	0.6198	6.3129
NCC-driven reconstruction	0.6495	0.4320	9.2430
Spine-DART	0.7461	0.6083	6.3431

Additionally, we evaluated Spine-DART on the *VerSe-small_random* dataset to assess reconstruction performance under more diverse viewing conditions, using arbitrary pairs of randomly sampled DRRs. A total of 1,153 vertebrae were successfully reconstructed, meeting the requirement that each view pair contains at least three predicted landmarks in common. Table 3 illustrates the reconstruction results on both the *VerSe-small_random* dataset and real X-rays from our cadaver specimen. Notably, 12 out of 16 vertebrae from the cadaver study were successfully reconstructed and annotated with transpedicular trajectories, providing an initial demonstration of applicability beyond purely synthetic data.

**TABLE 3:** 3D vertebra reconstruction performance of our approach using arbitrary-view X-rays. Results are shown on synthetic DRRs from the *VerSe-small_random* dataset and real clinical X-rays.

Dataset	DICE ↑	NSD ↑	HD-95 ↓
*VerSe-small_random*	0.7264	0.5671	7.3337
Real X-rays	0.7280	0.5231	7.1910

**FIGURE 7:**
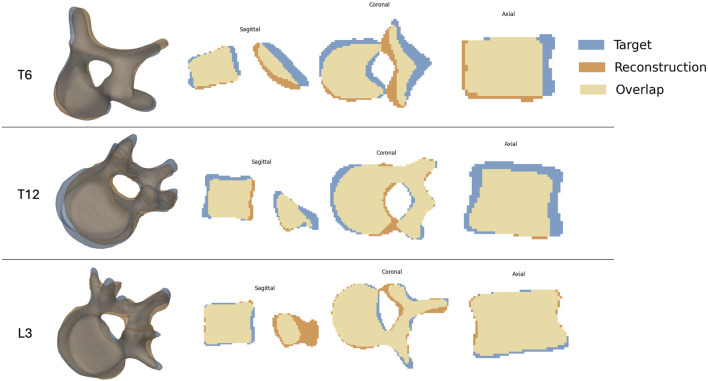
Qualitative results of three reconstructed vertebrae from Verse19 via Spine-DART from two random X-ray views.

Qualitative examples of the achieved reconstructions are shown in Figure 7.

### 4.4 Automatic transpedicular path planning

While vertebra reconstruction is an important intermediate step in our pipeline—enhancing interpretability and imposing anatomical constraints—the primary goal is to automatically plan surgical paths along the vertebral pedicles. To benchmark the performance of Spine-DART, we also implement a baseline method inspired by Killeen et al. (2023b), which performs 2D path planning followed by geometric triangulation to estimate the 3D trajectory.

The baseline model, which we will refer to as 2D-GeoPlan, is a DL model that shares the same architecture as our vertebra segmentation network—Mask R-CNN with a SWIN backbone (Chen et al., 2019)— and was also trained on DRRs generated from the CTSpine1K and NMDID datasets. Ground truth annotations were propagated using our SSM, limited to vertebrae that were successfully registered. Trajectory segmentations were defined in 3D as cylinders with a 5 mm diameter, following Baroud and Steffen (2005). To avoid using annotations with cortical breaches, we excluded trajectories whose minimum distance to the pedicle walls was less than 2.5 mm.

We applied 2D-GeoPlan to both the *VerSe-small_random* dataset and the real X-rays. As before, two views were randomly selected per sample, and the predicted paths were triangulated to generate the 3D cylindrical reconstructions. Using this approach, both transpedicular paths were successfully reconstructed for 928 out of 1,407 vertebrae in the *VerSe-small_random* dataset. For the real X-rays, full reconstruction of both paths was achieved in 5 out of 16 vertebrae.

**FIGURE 8:**
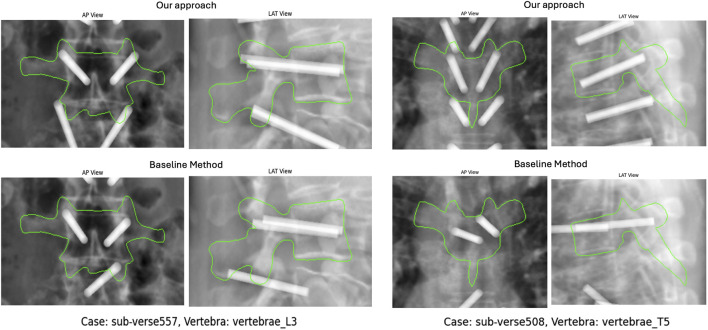
Anteroposterior (AP) and lateral (LAT) DRRs for two vertebral samples from VerSe19. Cannula placements are shown for 2D-GeoPlan (top) and Spine-DART (bottom).

**FIGURE 9:**
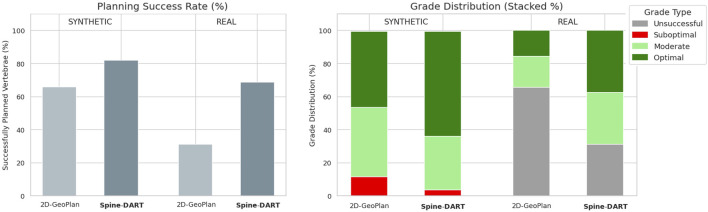
Left: Percentage of successfully planned vertebrae for the 2D-GeoPlan and **Spine-DART** models, shown separately for the synthetic dataset and the real X-ray dataset. Right: Stacked grade distribution of all evaluated cannula paths (200 synthetic paths and 32 real X-ray paths), illustrating the proportion of unsuccessful, suboptimal, moderate, and optimal plans assigned by a clinical expert. Together, the plots summarize overall planning success and qualitative path assessment for both models across synthetic and real data.

Since there is no single correct solution for the transpedicular trajectory, we did not directly compare the reconstructed paths to manual annotations. Instead, we randomly selected 100 vertebrae that had successful reconstructions from both the baseline and our proposed method. These reconstructed paths, along with those from a cadaver specimen, were evaluated by a medical resident in Interventional Radiology at Johns Hopkins School of Medicine. For the assessment, we simulated orthogonal DRRs with the reconstructed cannulas rendered as metallic objects within the scene (Figure 8). The clinician graded the placement accuracy of each cannula on a 3-point scale: 0 — Suboptimal, 1 — Moderate/requires adjustment, and 2 — Optimal placement. Figure 9 summarizes the grade distribution across the 100 vertebrae (200 cannulas in total) based on the expert’s evaluation.

### 4.5 Discussion and conclusion

Accurate 3D surgical planning from intraoperative 2D X-rays remains a key challenge for enabling robotic assistance in procedures where preoperative CT is not available. In this work, we presented Spine-DART, an optimization-based VA planning framework that enables CT-free 3D transpedicular path planning from only two intraoperative X-ray images. By combining statistical shape models (SSMs) with differentiable rendering, Spine-DART accommodates flexible, non-orthogonal imaging configurations while producing anatomically consistent reconstructions suitable for downstream planning.

Across synthetic benchmarks, Spine-DART achieves reconstruction accuracy comparable to state-of-the-art deep learning methods trained under fixed-view protocols. While data-driven approaches such as ReVerteR can achieve slightly better reconstruction metrics under idealized conditions, their reliance on fixed geometries and lack of explicit anatomical correspondences limit their utility for path planning. In contrast, Spine-DART trades minor decreases in reconstruction precision for greater flexibility, interpretability, and planning-readiness. Qualitative clinician-in-the-loop evaluation further suggests that the resulting plans are frequently clinically viable, outperforming a 2D–3D triangulation baseline in subjective assessment.

Beyond line-based planning, the availability of dense anatomical correspondences enables the propagation of complex, multi-segment trajectories, which are essential for advanced procedures such as curved Balloon Kyphoplasty. Moreover, the differentiable formulation naturally supports future extensions, including biomechanical constraints and multi-objective optimization to balance surgical access, safety margins, and implant positioning. While Spine-DART is demonstrated using only two X-rays, the framework can be readily extended to incorporate additional views when clinically available, further improving reconstruction accuracy and planning robustness.

Nevertheless, the pipeline has limitations. Its modular nature means that overall performance depends on the accuracy of several upstream components, including landmark detection, SSM fitting capability, and image similarity estimation. In practice, the most common failure mode arises when insufficient or inconsistent landmarks are detected across views, often due to occlusions, low contrast, or extreme viewing angles, which can compromise initialization. While backup strategies and semantic priors help mitigate this, incorporating probabilistic keypoint confidence or attention mechanisms could improve robustness. Less frequently, unfavorable bi-planar configurations with limited angular separation reduce geometric constraints and may lead to slower convergence or suboptimal solutions. An additional failure mode arises from the learned image similarity loss used during optimization. Because this loss model is trained on synthetic fitting examples, it may not fully capture the distribution of reconstruction states encountered during optimization, leading to suboptimal gradients and occasional convergence to visually plausible but geometrically inaccurate solutions. Future improvements will focus on strengthening the loss model itself, for example, by augmenting training data, incorporating geometry-aware supervision, or combining learned similarity with analytic overlap constraints.

Additionally, the very strength of the SSM—its ability to enforce anatomically plausible shapes—can limit reconstruction accuracy when fitting low-prevalence morphologies, such as compressed or fractured vertebrae, which are precisely the cases targeted by vertebral augmentation. In such scenarios, the model may bias reconstructions toward healthy anatomical priors, potentially under-representing fracture severity or local deformities. While this constraint can help prevent implausible solutions, it may reduce fidelity in regions most critical for accurate planning. Future work will therefore focus on improving shape modeling for these cases, including per-level vertebral representations and enriched training sets with pathological anatomy.

While we emphasize robustness to arbitrary bi-planar X-ray views, it is important to clarify that “arbitrary” refers to flexible, non-orthogonal view configurations acquired under a known and reasonably calibrated imaging geometry. As with most differentiable rendering–based approaches, Spine-DART assumes access to the projection parameters of the X-ray system. In our cadaver study performed using a LoopX (Brainlab) system, accurate projection matrices were available and enabled reliable optimization. Nevertheless, inaccuracies in geometric calibration could degrade reconstruction quality in broader clinical settings.

In addition, although the clinician-in-the-loop assessment provides valuable qualitative insight into the feasibility of the planned trajectories, it relies on subjective scoring. Future work could complement such evaluations with objective safety metrics, such as proximity to pedicle walls, cortical breach measures, or quantitative safety margins, to provide more standardized and reproducible assessment of planning quality.

Ultimately, this work demonstrates the feasibility of accurate 3D path planning for robot-assisted vertebroplasty from bi-plane X-rays using a differentiable rendering framework. By eliminating the need for preoperative CT and accommodating flexible intraoperative imaging configurations, Spine-DART serves as an enabling technology for CT-free planning under real-world constraints. While the proposed approach shows clear advantages over conventional baselines and promising results in an initial cadaver study, the current validation remains limited in scale, and broader evaluation on real clinical data will be required before claims regarding routine clinical deployment can be made.

## Data Availability

The raw data supporting the conclusions of this article will be made available by the authors, without undue reservation.
